# Prevalence and Prognostic Impact of Coronary Chronic Total Occlusions in Patients With Cardiogenic Shock

**DOI:** 10.1002/ccd.70116

**Published:** 2025-08-25

**Authors:** Michael Behnes, Moritz Schmidberger, Giuseppe Vadalà, Alice Moroni, Juan‐Luis Guiterrez‐Chico, Stylianos Pyxaras, Alexander Bufe, Florian Krötz, Kambis Mashayekhi, Mohamed Ayoub, Jonas Rusnak, Mohammad Abumayyaleh, Jonas Dudda, Kathrin Weidner, Ibrahim Akin, Tobias Schupp

**Affiliations:** ^1^ Department of Cardiology, Angiology, Haemostaseology and Medical Intensive Care, University Medical Centre Mannheim, Medical Faculty Mannheim Heidelberg University Baden‐Württemberg Germany; ^2^ Division of Cardiology University Hospital “P. Giaccone” Palermo Italy; ^3^ Division of Cardiology Imelda Hospital Bonheiden Belgium; ^4^ Bundeswehrzentralkrankenhaus Koblenz Germany; ^5^ Department of Cardiology Klinikum Fürth Fürth Germany; ^6^ Interventional Cardiology, HELIOS Klinikum Wuppertal, Herzzentrum Universität Witten/Herdecke Herdecke Germany; ^7^ Department of Cardiology Klinikum Starnberg Starnberg Germany; ^8^ Department of Internal Medicine and Cardiology Mediclin Heart Centre Lahr Lahr Germany; ^9^ Division of Cardiology and Angiology Heart Center University of Bochum‐Bad Oeynhausen Bad Oeynhausen Germany

**Keywords:** cardiogenic shock, chronic total occlusion, coronary artery disease, critical care, internal medicine

## Abstract

**Background:**

Mortality in patients with cardiogenic shock (CS) remains high despite advanced treatment strategies in CS patients, underlining the need for the identification of predictors of prognosis in CS patients. Therefore, the present study investigates the prognostic impact of coronary chronic total occlusions (CTO) in patients with CS.

**Methods:**

All consecutive patients being acutely admitted with CS to an intensive care unit (ICU) and undergoing invasive coronary angiography (ICA) from 2019 to 2021 were included, irrespective of the etiology of CS. Patients with at least one CTO were compared to non‐CTO patients with regard to the risk of all‐cause mortality at 30 days. Further risk stratification was performed according to the extent of coronary artery disease (CAD).

**Results:**

A total of 192 CS patients undergoing ICA during index hospitalization were included. At least one CTO was present in 24% of CS patients. Patients with CTO were older (median 78 vs. 68; *p* = 0.001) and presented more frequently with non‐ST‐elevated myocardial infarction (21% vs. 12%; *p* = 0.048). The presence of a CTO was associated with higher rates of 30‐days all‐cause mortality (70.2% vs. 47.6%; HR = 1.783, 95% CI 1.176−2.702; *p* = 0.009), even after multivariable adjustment (adjusted HR = 1.898; 95% CI 1.116−3.229; *p* = 0.018). Patients with CTO were accompanied by an even higher 30‐days all‐cause mortality as compared to patients with multi‐vessel CAD without CTO (adjusted HR = 1.723; 95% CI 1.058−2.805; *p* = 0.029).

**Conclusion:**

Coronary CTO are common in patients with CS and represent an independent predictor of all‐cause mortality at 30 days.

## 1 Introduction

Cardiogenic shock (CS) is characterized by impaired cardiac output resulting into life‐threatening hypoperfusion and end‐organ failure. Over the past decades, the rates of hospital admissions for CS have tripled, however, despite advances in the medical care for CS patients, CS is still accompanied by short‐term mortality rates of up to 50% at 30 days [1]. Traditionally, acute myocardial infarction (AMI) represented the most common cause of CS. As a consequence of improvements in AMI treatment and subsequent improved survival rates, including nationwide AMI care programs with appropriate timing of percutaneous coronary intervention (PCI), pharmacotherapies, and cardiac implantable electronic devices (CIED), the proportion of patients suffering from AMI‐related CS is decreasing. In contrast, the proportion of patients with CS due to progressive heart failure (HF) increases related to demographic changes and the outlined improved surival following AMI. Therefore the spectrum of CS in daily clinical practice has changed over time [1, 2, 3].

A large proportion of patients with CS suffers from coronary artery disease (CAD), with a corresponding prevalence exceeding 80% [4, 5]. Related to the presence of chronic coronary syndromes affecting patients with a long‐standing history of CAD, the complexity of CAD itself was demonstrated to increase, alongside with higher rates of complex coronary lesions located at the coronary ostia, the left main stem and severely diffused calcification [6, 7]. In line, the prevalence of coronary chronic total occlusions (CTO) was shown to increase to up to 25% of patients undergoing invasive coronary angiography (ICA), with studies reporting a near doubling prevalence within recent decades [8, 9, 10, 11]. Although several studies have identified the presence of CTO as an independent predictor of adverse prognosis in chronic HF, most studies have excluded critically ill patients and did not investigate the prognosis of CTO in such vulnerable subsets of patients [12, 13, 14]. Accordingly, the prognostic impact of CTO in patients with CS was investigated only within few studies that were usually restricted to patients with AMI‐related CS [7, 15].

Therefore, the present study sought to investigate the prognostic impact of CTO in patients with CS irrespective of the underlying CS etiology.

## 2 Methods

### 2.1 Study Patients, Design, and Data Collection

The single‐center registry included all consecutive patients presenting with CS of any cause, being acutely admitted to the intensive care unit (ICU) for internal medicine at Mannheim University Hospital from June 2019 to May 2021, as recently published [16]. Data collection was performed using the electronic hospital information system and the IntelliSpace Critical Care and Anesthesia Information System (ICCA, Philips, Philips GmbH Market DACH, Hamburg, Germany). Data relevant to the index event included admission documents, vital signs, laboratory values, treatment data, diagnostic imaging and information on the pharmacotherapy administered. Furthermore, important laboratory data, common ICU scores, hemodynamic measurements and ventilation parameters were recorded. The present study is derived from the prospective “Cardiogenic Shock Registry Mannheim” (CARESMA‐registry; clinicaltrials.gov identifier: NCT05575856). The study was conducted in accordance with the principles of the Declaration of Helsinki and approved by the medical ethics committee II of the Medical Faculty Mannheim, University of Heidelberg, Germany.

### 2.2 Inclusion and Exclusion Criteria

All consecutive patients with CS undergoing ICA during index hospitalization were included. The diagnosis of CS was determined in accordance with the current recommendations of the Acute Cardiovascular Care Association of the European Society of Cardiology [17]. Accordingly, CS was defined by persisting (> 30 min) hypotension (systolic blood pressure [SBP] < 90 mmHg) despite adequate filling status. Consistently, the need for vasopressor or inotropic therapy to achieve a SBP > 90 mmHg was included. In addition, all patients had to show signs of hypoperfusion of end organs, such as oliguria with a urine output < 30 mL/h, altered mental status, cold clammy skin, and elevated lactate > 2 mmol/L. For the present study, all patients with CS, irrespective of CS etiology were included. The underlying etiologies included AMI, ventricular tachyarrhythmias, acute decompensated HF, pulmonary embolism, valvular heart disease, aortic dissection, cardiomyopathies and pericardial tamponade. According to current international guidelines, ST segment myocardial infarction (STEMI) was defined as a novel rise in the ST segment in at least two contiguous leads with ST‐segment elevation of ≥ 2.5 mm in men younger than 40 years, of ≥ 2 mm in men 40 years of age or older, or of ≥ 1.5 mm in women in leads V2 and V3, 1 mm in the other leads, or both. NSTEMI was defined as the presence of an acute coronary syndrome with a troponin I increase above the 99th percentile of a healthy reference population in the absence of ST‐segment elevation, but persistent or transient ST‐segement depression, inversion, or alteration of T wave or normal ECG findings in the presence of a coronary culprit lesion [18]. Since cardiac tamponade and pulmonary embolism are defined as probable underlying causes of acute HF and CS in current guidelines and recommendations, they were identified as forms of CS despite the obstructive mode of shock [19, 20]. Aortic dissection was classified as CS, when aortic valve regurgitation was the primary source of hemodynamic instability [20]. The severity of CS was categorized according to the Society for Cardiovascular Angiography & Interventions (SCAI) shock classification [21]. In addition, the Synergy between PCI with TAXUS and Cardiac Surgery Score I (SYNTAX score I) was determined for all patients to assess the complexity and extent of CAD [22]. Patients without ICA during index hospitalization were excluded.

### 2.3 Definition of CTO

For the present study, all data related to ICA (i.e., imaging files and source data) were reviewed by two independent cardiologists. A coronary CTO was defined in accordance with the European consensus as a 100% stenosis with Thrombolysis In Myocardial Infarction (TIMI) 0 flow for more than 3 months [23]. The CTO group included patients with at least one CTO, including also patients with prior or acute revascularization of a non‐CTO vessel by PCI or coronary artery bypass grafting (CABG), and patients with an occluded bypass graft distal to the CTO. The non‐CTO group included all patients without CAD, CAD without CTO, CABG patients without a concomitant CTO (as defined above), as well as patients with successfully revascularized CTO before index hospitalization.

### 2.4 Study Endpoint

The study endpoint was defined as all‐cause mortality at 30 days (i.e. short‐term follow‐up). All‐cause mortality data was obtained using the electronic hospital information system and by directly contacting state resident registration offices (“bureau of mortality statistics”).

### 2.5 Statistical Methods

Quantitative data were presented as mean ± standard error of mean (SEM), median and interquartile range (IQR), and ranges depending on the distribution of the data and were compared using the Student's *t*‐test for normally distributed data or the Mann−Whitney *U* test for nonparametric data. Deviations from a Gaussian distribution were tested by the Kolmogorov−Smirnov test. Qualitative data were presented as absolute and relative frequencies and were compared using the Chi‐square test or the Fisher's exact test, as appropriate. Kaplan−Meier analyses were performed stratified by the presence or absence of CTO. Thereafter the prognosis of patients with CTO was compared to patients with non‐CTO, stratified by non‐CAD, single‐vessel CAD and multivessel CAD. Univariable hazard ratios (HR) were given together with 95% confidence intervals. The prognostic impact of CTO was thereafter investigated within multivariable Cox regression models and adjusted for age, sex, body mass index, diabetes mellitus, congestive HF, AMI, cardiopulmonary resuscitation, baseline lactate, baseline creatinine levels, and SYNTAX score I.

Results of all statistical tests were considered significant for *p* ≤ 0.05, a statistical trend was defined for p ≤ 0.10. The software SPSS (Version 28, IBM, Armonk, New York) was used for statistics.

## 3 Results

### 3.1 Study Population

**Figure 1:**
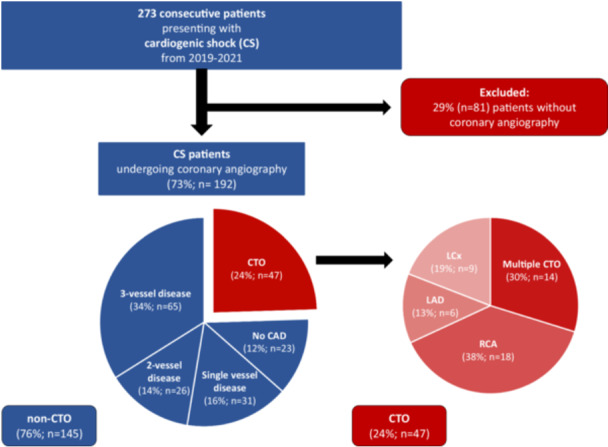
Flowchart cohort composition. [Color figure can be viewed at wileyonlinelibrary.com]

From June 2019 until May 2021, a total of 273 consecutive patients with CS were treated at our ICU for internal medicine. Overall, 73% of patients underwent ICA during index hospitalization, resulting in 192 CS patients eligible for the study. As outlined in Figure 1, the corresponding CTO rate was 24% (*n* = 47; CTO‐group) and 76% (*n* = 145) of patients were allocated to the non‐CTO group.

Patients with at least one CTO were older compared to patients without (median 78 vs. 68 years; *p* = 0.001) (Table 1). In contrast, the distribution of sex, cardiovascular risk profile and prior medical history did not significantly differ among CTO and non‐CTO patients, except for higher rates of prior CAD in CTO patients (51% vs. 32%; *p* = 0.001). Patients with CTO were admitted with a lower heart rate (median 71 vs. 90 bpm; *p* = 0.006) and lower SBP (median 104 vs. 115 mmHg; *p* = 0.032) compared to non‐CTO patients. The medication before admission did not significantly differ between both groups, except for a higher use of beta blockers in the CTO group (55% vs. 38%; *p* = 0.042). Baseline laboratory values on admission revealed higher creatinine levels in CTO compared to non‐CTO patients (median 1.5 vs. 1.3 mg/dL; *p* = 0.006), whereas pH, lactate and cardiac troponin I levels were comparable between the two groups.

**Table 1:** Baseline characteristics.

	Non‐CTO (*n* = 145)		CTO (*n* = 47)		*p* value
Age, median (IQR)	68	(59−78)	78	(71−82)	**0.001**
Male sex, *n* (%)	90	(62.1)	30	(63.8)	0.864
Body mass index, kg/m^2^, median (IQR)	26.2	(23.9−29.5)	26.2	(24.7−29.4)	0.376
Vital signs, median; (IQR)					
Body temperature (°C)	36.0	(34.8−36.6)	36.0	(34.8−36.5)	0.519
Heart rate (bpm)	90	(73−105)	71	(61−97)	**0.006**
Systolic blood pressure (mmHg)	115	(99−134)	104	(84−126)	**0.032**
Respiratory rate (breaths/min)	19	(17−22)	19	(16−23)	0.658
Cardiovascular risk factors, *n* (%)					
Arterial hypertension	98	(67.6)	38	(80.9)	0.098
Diabetes mellitus	48	(33.1)	22	(46.8)	0.116
Hyperlipidemia	73	(50.3)	26	(55.3)	0.616
Smoking	53	(36.6)	15	(31.9)	0.603
Prior medical history, *n* (%)					
Coronary artery disease	46	(31.7)	24	(51.1)	**0.001**
Congestive heart failure	38	(26.2)	17	(36.2)	0.198
Atrial fibrillation	35	(24.1)	13	(27.2)	0.699
Chronic kidney disease	35	(24.1)	16	(34.0)	0.189
Stroke	16	(11.0)	7	(14.9)	0.451
COPD	21	(14.5)	6	(12.8)	1.000
Liver cirrhosis	5	(3.4)	1	(2.1)	1.000
Medication on admission, *n* (%)					
ACE‐inhibitor	44	(30.3)	19	(40.4)	0.215
AT‐1 antagonist	22	(15.2)	7	(14.9)	1.000
Beta‐blocker	55	(37.9)	26	(55.3)	**0.042**
ARNI	3	(2.1)	2	(4.3)	0.598
Aldosterone antagonist	13	(9.0)	9	(19.1)	0.068
Diuretics	49	(33.8)	21	(44.7)	0.222
ASA	39	(26.9)	16	(34.0)	0.358
P2Y12‐inhibitor	13	(9.0)	6	(12.8)	0.415
Statin	55	(37.9)	19	(40.4)	0.863
Baseline laboratory values Day 1, median (IQR)					
pH	7.29 (7.22−7.36)		7.29 (7.19−7.36)		0.613
Lactate, mmol/L	3.0 (1.7−6.1)		3.3 (1.6−7.5)		0.703
Sodium mmol/L	138 (136−141)		13 7(135−140)		0.202
Potassium, mmol/L	4.2 (3.7−4.7)		4.4 (3.9−4.9)		0.299
Creatinine, mg/dL	1.3 (1.0−1.8)		1.5 (1.3‐2.3)		**0.006**
Hemoglobin, g/dL	12.9 (11.2−14.2)		12.5 (10.3−13.6)		0.090
WBC count, × 10^9^/L	15.9 (10.7−19.6)		13.5 (10.2−18.2)		0.194
Platelet count, × 10^9^/L	225.0 (175.5−282.5)		226.5 (188.5−259.5)		0.723
Troponin I, μg/L	1.420 (0.223−11.503)		1.771 (0.262−9.987)		0.090
NTproBNP, pg/L	1786 (327−10,971)		5270 (1164−27,225)		0.077

*Note:* Level of significance *p* < 0.05. Bold type indicates statistical significance.

Abbreviations: ACE, angiotensin‐converting enzyme; ARNI, angiotensin‐receptor‐neprilysin‐inhibitor; ASA, acetylsalicylic acid; AT‐1, angiotensin II receptor type 1; COPD, chronic obstructive pulmonary disease; CTO, chronic total occlusion; IQR, interquartile range; INR, international normalized ratio; NT‐pro BNP, amino‐terminal pro‐B‐type natriuretic peptide; P2Y12, purinergic receptor P2Y G‐protein coupled 12 protein; WBC, white blood cells.

CS‐related data are summarized in Table 2. AMI was the leading cause of CS within the entire study cohort (67%), followed by acute decompensated HF (13%), arrhythmias (12%), cardiomyopathies (3%). The distribution of CS etiologies did not differ significantly between the CTO and non‐CTO group, except for higher rates of NSTEMI (21% vs. 12%, *p* = 0.048) in CTO patients and STEMI in non‐CTO patients (53% vs. 49%, *p* = 0.048). Within the entire cohort, 64% of patients (*n* = 123) suffered from cardiac arrest, with comparable rates between CTO and non‐CTO patients (overall: 64% vs. 65%; out‐of‐hospital: 43%; in‐hospital: 21%; *p* = 0.975). Similarly, the need for mechanical ventilation (55% vs. 61%, *p* = 0.496) and mechanical circulatory support devices (11% vs. 12%; *p* = 1.000) did not differ significantly between the two groups. Furthermore, baseline echocardiographic data were comparable between CTO and non‐CTO patients.

**Table 2:** Shock related data.

	Non‐CTO (*n* = 145)		CTO (*n* = 47)		*p* value
Cause of CS, *n* (%)					
Acute myocardial infarction	95	(65.5)	33	(70.2)	0.469
Arrhythmic	15	(10.3)	8	(17.0)	
Acute decompensated heart failure	20	(13.8)	5	(10.6)	
Pulmonary embolism	1	(0.7)	0	(0.0)	
Valvular heart diseases	5	(3.4)	0	(0.0)	
Cardiomyopathy	6	(4.1)	0	(0.0)	
Pericardial tamponade	3	(2.1)	1	(2.1)	
Myocardial infarction, *n* (%)					
No myocardial infarction	50	(34.5)	14	(29.8)	**0.048**
STEMI	77	(53.1)	23	(48.9)	
NSTEMI	18	(12.4)	10	(21.3)	
Classification of CS,^a^ *n* (%)					
Stage A	0	(0.0)	0	(0.0)	0.999
Stage B	3	(2.1)	1	(2.1)	
Stage C	39	(26.9)	13	(27.7)	
Stage D	10	(6.9)	3	(6.4)	
Stage E	93	(64.1)	30	(63.8)	
Transthoracic echocardiography					
LVEF > 55%, *n* (%)	12	(8.3)	3	(6.4)	0.798
LVEF 54%−41%, *n* (%)	17	(11.7)	3	(6.4)	
LVEF 40%−30%, *n* (%)	37	(25.5)	12	(25.5)	
LVEF < 30%, *n* (%)	68	(46.9)	24	(51.1)	
LVEF not documented, *n* (%)	11	(7.5)	5	(10.6)	
TAPSE (mm), median (IQR)	17	(13−20)	14	(11−20)	0.368
Cardiopulmonary resuscitation					
Out‐of‐hospital cardiac arrest, *n* (%)	64	(44.1)	20	(42.6)	0.975
In‐hospital cardiac arrest, *n* (%)	29	(20.9)	10	(21.3)	
Shockable rhythm, *n* (%)	56	(38.6)	16	(34.0)	0.607
ROSC (min), median (IQR)	14	(8−28)	15	(12−20)	0.643
Respiratory status					
Mechanical ventilation, *n* (%)	89	(61.4)	26	(55.3)	0.496
Duration of Mechanical ventilation, days, mean (IQR)	2	(1−6)	2	(0−5)	0.370
PaO_2_/FiO_2_ ratio, median; (IQR)	222	(142−331)	305	(152−367)	0.215
PaO_2_, mmHg, median (IQR)	109	(78−172)	131	(81−166)	0.662
Multiple organ support					
Norepinephrine dose on admission (μg/kg/min), median; (IQR)	0.1	(0.0−0.3)	0.1	(0.0−0.2)	**0.043**
Mechanical circulatory assist device, *n* (%)	18	(12.4)	5	(10.6)	1.000
Follow‐up data, median (IQR)					
ICU time (days)	4	(2−8)	3	(1−6)	0.067
Follow‐up time (days)	31	(2−31)	4	(1−31)	**0.001**

*Note:* Level of significance *p* < 0.05. Bold type indicates statistical significance.

Abbreviations: CS, cardiogenic shock; CTO, chronic total occlusion; FiO_2_, fraction of inspired oxgen; ICU, intensive care unit; IQR, interquartile range; LVEF, left ventricular ejection fraction; PaO2, partial pressure of oxygen; ROSC, return of spontaneous circulation; TAPSE, tricuspid annular plane systolic excursion.

^a^ SCAI SHOCK stage classification.

### 3.2 Angiographic and CTO‐Related Characteristics

In the entire study cohort the overall CAD rate was 88%. As outlined in Table 3, patients with CTO had higher rates of multi‐vessel CAD (94% vs. 63%; *p* = 0.001) and specifically 3‐vessel CAD (81% vs. 45%; *p* = 0.001) compared to non‐CTO patients. In line with these findings, the anatomical complexity of CAD, as assessed by the SYNTAX score I, was significantly greater in the CTO group with a median score of 35.0 (IQR 24.0−44.0) compared to 12 (IQR 4.5−25.0; *p* = 0.001) in non‐CTO patients. The right coronary artery (RCA) was the most frequently diseased vessel in CTO compared to non‐CTO patients (83% vs. 39%; *p* = 0.001). Coronary revascularizations were performed frequently in both groups, with similar PCI rates (66% vs. 68%; *p* = 0.768), whereas CTO patients were more frequently referred for emergency or subsequent CABG surgery (23% vs. 5%; *p* = 0.001). Overall, CTO‐PCI was only performed in 9 patients (19%) during index hospitalization with a corresponding success rate of 89%. In the CTO group, most patients had a CTO of the RCA (38%), followed by the left circumflex artery (LCx; 19%) and the left artery descending (LAD; 13%). Notably, 30% of CTO patients suffered from multiple CTO.

**Table 3:** Angiographic and CTO characteristics.

	Non‐CTO (*n* = 145)		CTO (*n* = 47)		*p* value
CAD types, *n* (%)					
No evidence of CAD	23	(15.9)	0	(0.0)	**0.001**
Single‐vessel disease	31	(21.4)	3	(6.4)	
Two‐vessel disease	26	(17.9)	6	(12.8)	
Three‐vessel disease	65	(44.8)	38	(80.9)	
Coronary revascularization, *n* (%)					
PCI	99	(68.3)	31	(66.0)	0.768
Sent to CABG	7	(4.8)	11	(23.4)	**0.001**
Affected vessel, *n* (%)					
RCA	57	(39.3)	39	(83.0)	**0.001**
LMT	19	(13.1)	4	(8.5)	0.339
LAD	75	(51.7)	35	(74.5)	**0.006**
LCx	61	(42.1)	35	(74.5)	**0.001**
SYNTAX‐Score, median (IQR)	12	(4.5−25.0)	35	(24.0−44.0)	**0.001**
CTO specific data^a^					
Affected CTO vessel, *n* (%)					
RCA	—	—	18	(38.3)	—
LAD	—	—	6	(12.8)	—
LCx	—	—	9	(19.1)	—
Occluded CABG on CTO vessel	—	—	9	(19.1)	—
Multiple CTO	—	—	14	(29.8)	—
CTO revascularization, *n* (%)					
CTO‐PCI	—	—	9	(19.1)	—
Succesfull CTO‐PCI	—	—	8	(88.9)	—
CTO lesion characteristics, *n* (%)					
CTO‐leng th ≥ 20 mm	—	—	32	(68.1)	—
Severe calcification	—	—	17	(36.2)	—
Bending > 45°	—	—	5	(10.6)	—
J‐CTO score ≥ 3	—	—	5	(10.6)	—
Poor distal vessel quality	—	—	28	(59.6)	—
Morphology of proximal stump, *n* (%)					
Tapered	—	—	24	(51.1)	—
Blunt	—	—	14	(29.8)	—
Ambigious	—	—	10	(21.3)	—
In‐Stent	—	—	0	(0.0)	—
Ostial	—	—	1	(2.1)	—
Type of collateralisation, *n* (%)					
Bridging	—	—	7	(14.9)	—
Ipsilateral	—	—	20	(42.6)	—
Contralateral	—	—	32	(68.1)	—
Epicardial	—	—	11	(23.4)	—
Intramyocardial	—	—	40	(85.1)	—
Werner classification, *n* (%)					
CC0	—	—	2	(4.3)	—
CC1	—	—	16	(34.0)	—
CC2	—	—	29	(61.7)	—

*Note:* Level of significance *p* < 0.05. Bold type indicates statistical significance.

Abbreviations: CABG, coronary artery bypass grafting; CAD, coronary artery disease; CTO, chronic total occlusion; LAD, left artery descending; LCx, left circumflex artery; LMT, left main trunk; (N)STEMI, (non)‐ST‐segment elevation myocardial infarction; PCI, percutaneous coronary intervention; RCA, right coronary artery; SYNTAX, SYNergy between TAXus and Cardiac Surgery; Werner Classification CC0, no continuous connection; CC1, threadlike connections; CC2, side‐branch like connections.

^a^ The CTO specific characteristics are only applicable for the CTO subgroup.

### 3.3 Prognostic Impact CTO in Patients With CS

**Figure 2:**
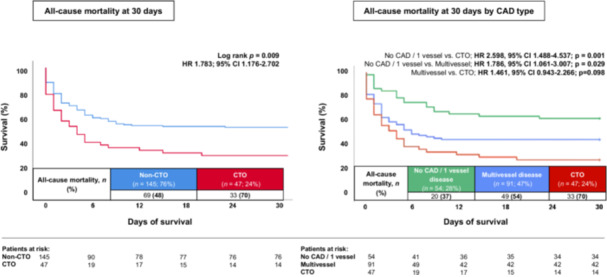
Kaplan−Meier survival analysis demonstrating: (left panel) 30‐day all‐cause mortality CTO versus non‐CTO; (right panel) 30‐day all‐cause mortality by extend of CAD. [Color figure can be viewed at wileyonlinelibrary.com]

In patients with CS, the presence of a coronary CTO was associated with a higher risk of 30‐days all‐cause mortality compared to non‐CTO patients (70% vs. 48%; HR = 1.783; 95% CI 1.176−2.702; *p* = 0.001; Figure 2A). When stratified by the extend of CAD, the highest risk of 30‐days all‐cause mortality was observed in CTO patients (70%), followed by multi‐vessel non‐CTO CAD patients (54%) and single‐vessel non‐CTO/non‐CAD patients (37%) (Figure 2B). Exemplarily, CTO patients revealed the highest risk of 30‐days all‐cause mortality compared to non‐CTO single vessel disease/non‐CAD patients (HR 2.598, 95% CI 1.488–4.537; *p* = 0.001). This association remained significant after excluding non‐CAD patients from the non‐CTO group (HR 1.552, 95% CI 1.021–2.364; *p* = 0.042). Furthermore, a statistical trend for a higher risk of 30‐days all‐cause‐mortality was observed for CTO patients compared to non‐CTO MVD patients (HR 1.461, 95% CI 0.943–2.266; *p* = 0.098).

After multivariable adjustment, including the SYNTAX score I to account for the anatomical complexity of CAD, the presence of at least one CTO was associated with higher 30‐days all‐cause mortality compared to non‐CTO patients (HR = 1.898; 95% CI 1.116−3.229; *p* = 0.018; Table 4). Furthermore, the presence of AMI (HR = 2.082; 95% CI 1.231−3.519; *p* = 0.006), cardiopulmonary resuscitation (HR = 1.553; 95% CI 1.126−2.143; *p* = 0.007), elevated levels of lactate at baseline (HR = 1.118; 95% CI 1.067−1.172; *p* = 0.001, per 1 mmol/L increase) and creatinine (HR = 1.201; 95% CI 1.010−1.428; *p* = 0.038; per 1 mg/dL increase) were independent predictors of short‐term all‐cause mortality. Finally, the presence of a CTO was associated with a higher risk of 30‐days all‐cause mortality even when compared to MVD patients (HR 1.723, 95% CI 1.058–2.805; *p* = 0.029) and after multivariable adjustment (not shown).

**Table 4:** Multivariable Cox regression analysis regarding all‐cause mortality at 30 days.

	HR	95% CI	*p* value
Age (per 1 year increase)	1.002	0.985−1.020	0.792
Male sex	1.583	0.997−2.514	0.051
Body mass index (per 1 kg/m^2^ increase)	1.017	0.967−1.070	0.506
Diabetes mellitus	1.028	0.633−1.68	0.912
Congestive heart failure	0.700	0.403−1.216	0.205
Acute myocardial infarction	2.082	1.231−3.519	**0.006**
Cardiopulmonary resuscitation	1.553	1.126−2.143	**0.007**
Baseline lactate (per 1 mmol/L increase)	1.118	1.067−1.172	**0.001**
Baseline creatinine (per 1 mg/dL increase)	1.201	1.010−1.428	**0.038**
SYNTAX score I (per 1 point increase)	1.001	0.984−1.019	0.866
CTO	1.898	1.116−3.229	**0.018**

*Note:* Level of significance *p* < 0.05. Bold type indicates statistical significance.

Abbreviations: CI, confidence interval; CTO, chronic total occlusion; HR, hazard ratio; ICU, intensive care unit; IQR, interquartile range; SYNTAX, Synergy between Percutaneous Coronary Intervention with TAXUS and Cardiac Surgery.

To assess the robustness of these findings, we conducted a sensitivity analysis excluding patients with non‐AMI etiology. Also in this AMI‐only cohort, CTO remained significantly associated with increased 30‐days all‐cause mortality after multivariable adjustment (HR = 1.832; 95% CI 1.013–3.314; *p* = 0.045; Supplemental Table 1). This association was further confirmed by Kaplan–Meier analysis (HR = 1.773; 95% CI 1.114–2.823; *p* = 0.016; Supporting Information S1: Figure 1).

## 4 Discussion

The present study sought to investigate the prognostic impact of coronary CTO in patients with CS, irrespective of the underlying etiology. At least one CTO was present in 24% of CS patients undergoing ICA. Patients with CTO were older, had higher rates of multi‐vessel CAD and were more frequently admitted with NSTEMI. The presence of a CTO was associated with an increased risk of short‐term all‐cause mortality at 30 days, even after multivariable adjustment. Furthermore, the presence of CTO was associated with impaired prognosis even when compared to patients with non‐CTO multi‐vessel CAD and adjusted for the complexity of CAD.

In line with previous findings, CTO patients in the present study were markedly older and presented with a higher extend of CAD, which may contribute to poorer outcomes as compared to non‐CTO patients [15, 24]. This may contribute to reduced coronary reserve, increasing vulnerability to both absolute and relative myocardial undersupply—regardless of the underlying CS etiology (i.e., STEMI, NSTEMI, and decompensated HF). Focussing on patients with MVD, the presence of non‐infarct related CTO (non‐IRA) has been shown to result in relevant supply‐demand imbalances deteriorating the prognosis of CTO‐MVD patients [11, 25]. Furthermore, previous data suggested a higher risk for the recurrence of life‐threatening ventricular tachyarrhythmias, as well as higher rates of all‐cause mortality in CTO patients [26, 27]. In the latter registry even the presence of multiple CTOs was a strong predictor of early cardiac death within 24 h after onset of ventricular tachyarrhythmias [26]. These findings highlight that patients with coronary CTO constitute a particular high‐risk subgroup within the broader CS population, characterized by advanced age, advanced comorbidities, and an increased susceptibility to multisystem organ failure, which is of major importance given ongoing demographic changes. Nevertheless, the presence of CTO was independently associated with an increased risk of all‐cause mortality in CS patients, even after multivariable adjustment and accompanied by highest short‐term mortality rates, even after excluding patients without CAD.

This is of major importance given the very high prevalence of CTO in almost one out of four patients (i.e., 24%) with CS, which was even higher compared to contemporary studies [8, 9, 28]. For instance, this prevalence exceeds the generally reported CTO rates in patients undergoing ICA (i.e., 11%) and in the general CAD population (i.e., 16%−19%) [14, 29]. Large registry data from the Swedish CTO registry suggests that CTO is a common risk factor for increased mortality in general populations, with the lowest attributable risk in patients with stable angina and higher risk in patients with STEMI [13]. Additionally CTO have already been identified as an independent risk factor in AMI complicated by CS [9, 11]. For instance, the CREDO‐Kyoto AMI registry demonstrated significantly higher rates of all‐cause mortality at both 30‐days and 5‐years of follow‐up in STEMI patients with multivessel disease (*n* = 313) complicated by CS and concomitant CTO, compared to those without CTO [11]. Similarily, a large retrospective cohort study based on the U.S. national inpatient sample included patients with AMI‐related CS undergoing ICA (*n* = 163,62). It was demonstrated that concomitant CTO were associated with adverse in‐hospital outcomes both in STEMI and NSTEMI patients. Notably, this analysis revealed a steadily increasing prevalence of coronary CTO during the 7‐year study period (2008–2014), rising from 10% to 21% [10].

The present study identifies coronary CTO as a strong and independent risk factor for 30‐days all‐cause mortality, irrespective of the underlying CS etiology. This is of utmost importance given the ongoing changes in the spectrum of CS. Shifts in patient demographics and comorbidities, along with advances in CS‐related therapies have contributed to a decline in the incidence of AMI and concurrent rise in the prevalence of HF. According to recent German registry data (*n* = 441,696), the incidence of CS increased from 33 per 100,000 in 2005 to 52 per 100,000 in 2017, while the proportion of cases attributable to AMI declined from 53% to 44% during the same period [30]. Non‐AMI‐CS even outnumbered AMI‐related CS in several studies, highlighting the changing epidemiology to increasingly include de novo or acute on chronic HF‐related CS [1, 30, 31]. As such, identifying prognostic markers and therapeutic strategies tailored to this expanding patient group is crucial. Given that coronary CTO has already been recognized as an independent risk factor in patients with chronic HF, these overlapping risk profiles further emphasize the rising clinical relevance of the interaction between CTO, HF, and CS in today's patient populations [12].

Current evidence remains inconclusive whether the poorer prognosis observed in CAD patients with CS is primarily driven by the overall extent of multi‐vessel CAD or by the presence of a coronary CTO itself. A study by Hoebers et al. including 609 patients with CS following STEMI suggested an association of multi‐vessel CAD and increased 30‐days all‐cause mortality, but did not identify the presence of CTO as an independent predictor of adverse prognosis [32]. In contrast, our study—based on a broader unselected CS cohort—demonstrated a significantly higher 30‐days mortality among patients with coronary CTO compared to those without. Importantly, this association was still evident after multivariable adjustment, identifying the presence of a CTO as an independent predictor of adverse outcome. Notably, mortality in patients with CTO exceeded that of patients with MVD but without CTO, suggesting that the increased risk is not merely a reflection of the extent of CAD, but rather underscores the specific and additive negative prognostic impact of a coronary CTO. Importantly, even after adjustment for the SYNTAX score I, the presence of a CTO remained an independent predictor of short‐term mortality. This finding supports the notion that CTO conveys specific prognostic information beyond the extent and complexity of CAD burden alone. Considering the shift in CS etiologies and the increasing incidence of CTO, its presence should be integrated into early risk stratification and clinical decision‐making. Recently, a substudy of the CULPRIT‐SHOCK trial suggested lower risk of death or renal replacement therapy in 157 AMI‐CS patients undergoing PCI of the culprit lesion only as compared to immediate complete revascularization [8]. Therefore, CTO‐PCI was not recommended during CS, but may become of prognostic value after survival and hemodynamic stabilization within a staged procedure.

As CTO become more prevalent in aging, comorbid patient populations, the need for further studies grows to define optimal management strategies for this high‐risk subgroup.

## 5 Study Limitations

Several limitations need to be acknowledged for the present study. The overall sample size was modest and no further risk stratification was performed according to CS etiology. Related to the single‐center study design we cannot exclude selection bias due to measurable and non‐measurable confounders. The proportion of patients undergoing CTO‐PCI was only modest (*n* = 9) and no further risk stratification was performed for patients undergoing CTO‐PCI.

## 6 Conclusion

Coronary CTO are found in up to 24% of patients presenting with CS of any type. The presence of CTO deteriorates the short‐term prognosis and is the strongest CAD‐related predictor of all‐cause mortality in CS at 30 days. CTO patients experience highest mortality‐rates compared to all other forms of CAD, comprising non‐CTO non‐CAD, non‐CTO single‐vessel CAD, or non‐CTO MVD.

## Conflicts of Interest

The authors declare no conflicts of interest.

## Supporting information


**Supplemental Table 1:** Multivariable Cox regression analysis regarding all‐cause mortality at 30 days after excluding non‐AMI patients.

Supplemental_figure1.
