# Supplementary material for: Prevalence and Prognostic Impact of Coronary Chronic Total Occlusions in Patients With Cardiogenic Shock
Source: Catheter Cardiovasc Interv. 2025 Aug 25;106(5):2805–15. doi: 10.1002/ccd.70116 (PMC12584578; doi:10.1002/ccd.70116)
Supplement: Supplementary file 1 — Supplemental Table 1: Multivariable Cox regression analysis regarding all‐cause mortality at 30 days after excluding non‐AMI patients. [file CCD-106-2805-s001.docx]

| **Supplemental Table 1. Multivariable Cox regression analysis regarding all-cause mortality at 30 days after excluding non-AMI patients.** | | | |
| --- | --- | --- | --- |
|  | **HR** | **95% CI** | **p value** |
| Age (per 1 year increase) | 1.005 | 0.983-1.027 | 0.676 |
| Male sex | 1.296 | 0.735-2.283 | 0.370 |
| Body mass index (per 1kg/m^2^ increase) | 1.026 | 0.958-1.098 | 0.467 |
| Diabetes mellitus | 1.275 | 0.724-2.247 | 0.400 |
| Congestive heart failure | 0.641 | 0.309-1.330 | 0.232 |
| Cardiopulmonary resuscitation | 1.311 | 0.904-1.901 | 0.153 |
| Baseline lactate (per 1 mmol/L increase) | 1.116 | 1.057-1.178 | **0.001** |
| Baseline creatinine (per 1 mg/dL increase) | 1.191 | 0.972-1.459 | 0.091 |
| SYNTAX-Score (per 1 point increase) | 1.006 | 0.986-1.026 | 0.579 |
| CTO | 1.832 | 1.013-3.314 | **0.045** |
| CI, confidence interval; CTO, chronic total occlusion; HR, hazard ratio; ICU, intensive care unit; IQR, interquartile range; SYNTAX, SYNergy between TAXus and Cardiac Surgery.  Level of significance p<0.05. Bold type indicates statistical significance. | | | |
