# Supplementary material for: Prevalence and Prognostic Impact of Coronary Chronic Total Occlusions in Patients With Cardiogenic Shock
Source: Catheter Cardiovasc Interv. 2025 Aug 25;106(5):2805–15. doi: 10.1002/ccd.70116 (PMC12584578; doi:10.1002/ccd.70116)
Supplement: Supplementary file 2 — Supplemental_figure1. [file CCD-106-2805-s002.pptx]

## Slide 1
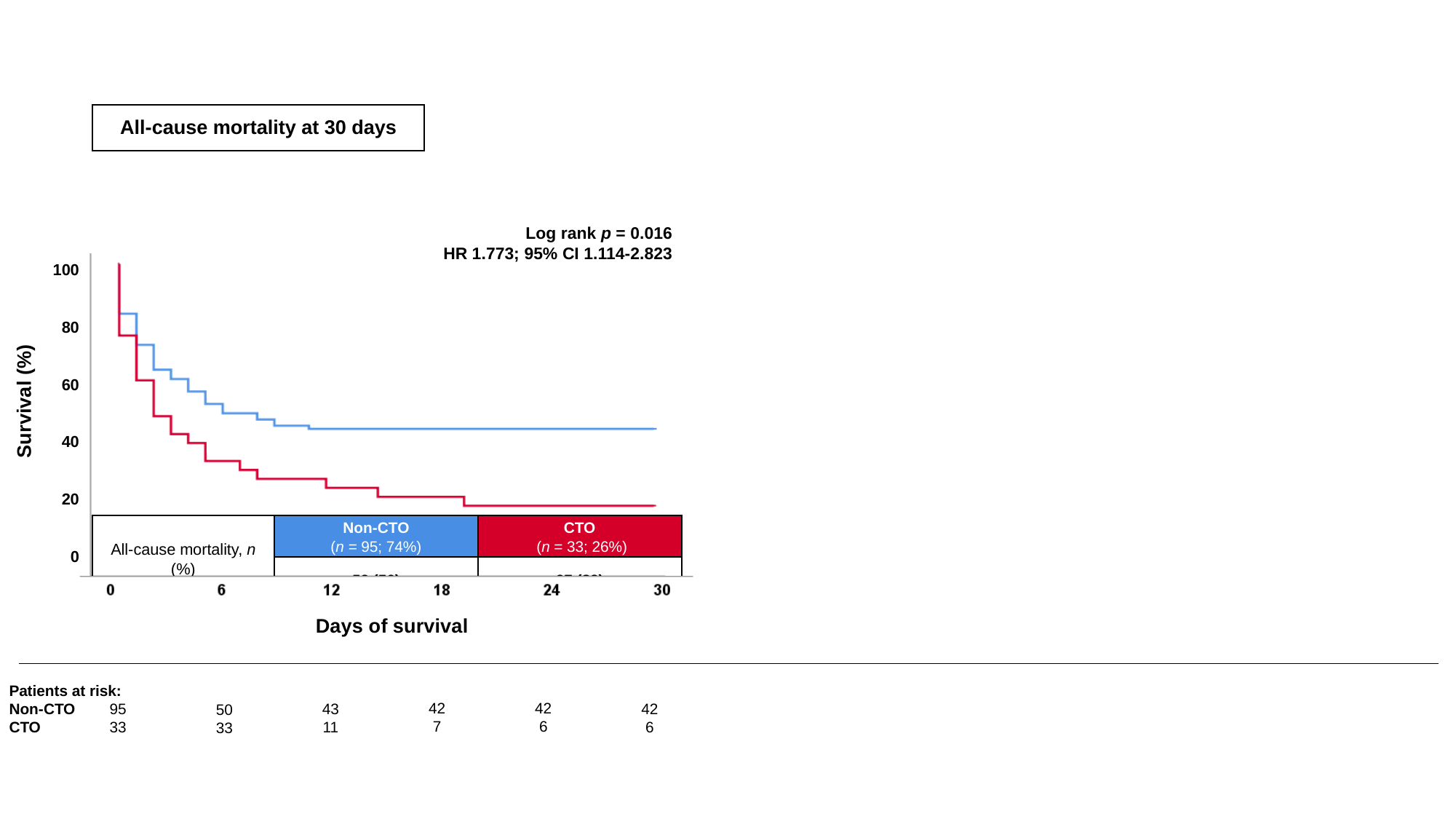

| All-cause mortality at 30 days |
| --- |
Log rank p = 0.016
HR 1.773; 95% CI 1.114-2.823
100
80
60
40
20
0
Survival (%)
| All-cause mortality, n (%) | Non-CTO (n = 95; 74%) | CTO (n = 33; 26%) |
| --- | --- | --- |
| | 53 (56) | 27 (82) |
Days of survival
Patients at risk:
Non-CTO
CTO
42
7
42
6
43
11
42
6
95
33
50
33
